# Analysis and Hardening of SEGR in Trench VDMOS with Termination Structure

**DOI:** 10.3390/mi14030688

**Published:** 2023-03-20

**Authors:** Yuan Wang, Tao Liu, Lingli Qian, Hao Wu, Yiren Yu, Jingyu Tao, Zijun Cheng, Shengdong Hu

**Affiliations:** 1 School of Microelectronics and Communication Engineering, Chongqing University, Chongqing 400044, China; 2 Science and Technology on Analog Integrated Circuit Laboratory, Chongqing 401332, China

**Keywords:** single-event gate-rupture (SEGR), heavy ion, trench VDMOS, radiation hardness

## Abstract

Single-event gate-rupture (SEGR) in the trench vertical double-diffused power MOSFET (VDMOS) occurs at a critical bias voltage during heavy-ion experiments. Fault analysis demonstrates that the hot spot is located at the termination of the VDMOS, and the gate oxide in the termination region has been damaged. The SEGR-hardened termination with multiple implantation regions is proposed and simulated using the Sentaurus TCAD. The multiple implantation regions are introduced, leading to an increase in the distance between the gate oxide and the hole accumulation region, as well as a decrease in the resistivity of the hole conductive path. This approach is effective in reducing the electric field of the gate oxide to below the calculated critical field, and results in a lower electric field than the conventional termination.

## 1. Introduction

Trench vertical double-diffused power MOSFETs (VDMOS) are widely used in power management systems as ideal power switches for space radiation environments [1,2,3]. These devices will be exposed to energetic neutrons, protons, and heavy ions when used in space. Heavy ions can trigger single-event gate-rupture (SEGR) in devices, causing gate oxide damage and even device burnout. Under the heavy-ion strikes, a large number of electron–hole pairs are generated along the track, and the gate oxide will break down to produce a conductive path, leading to a serious failure.

SEGR was first reported by Fischer [4], and since then, many studies have been carried out on the mechanism of SEGR. Darwish et al. [5] proposed that the surface electric field is sufficiently high to cause SEGR and that increasing the thickness of the gate oxide is an effective method to avoid SEGR. Nichols et al. [6] proved that SEGR is sensitive to the temperature and the incident angle. Titus et al. [7,8,9,10,11] studied the impact of particle energy, the position of particle Bragg peak, and the particle type on SEGR using experimental and simulation methods. Lauenstein et al. [12] reported the effects of charge generated by heavy ions in the gate oxide layer, the drift layer, and the substrate layer on SEGR. Titus et al. [13] reviewed the mechanism of single-event burnout (SEB) and SEGR. Privat et al. [14] investigated the impact of the latent defects on SEGR during switching operations. Recently, more studies have focused on the SEGR hardening technologies, such as the thicker gate oxide at the trench bottom proposed by Ttius et al. [3] and Darwish et al. [15], the W-shaped gate dielectric proposed by [16], the high-k gate dielectric proposed by X. Wan et al. [17] and A. Javanainen et al. [18], the widened split gate proposed by Jiang Lu et al. [19], the super-junction power MOS proposed by Muthuseenu et al. [20], and so on. As we all know, the termination is an essential part for all VDMOS devices; however, many hardening guidelines focus on the cell region of the VDMOS. The hardening technology for the termination is of great importance.

In this paper, heavy-ion tests were performed on trench VDMOS with a *V*_BR_ of 60 V. It was found that SEGR occurred in the termination and the gate oxide was damaged. The failure mechanism was analyzed using Sentaurus TCAD. The termination is more prone to SEGR than the cell, and thus, the SEGR-hardened termination with multiple implantation regions was proposed. It was shown to be an effective method to suppress SEGR and improve radiation reliability.

## 2. Device and Experiments

**Figure 1:**
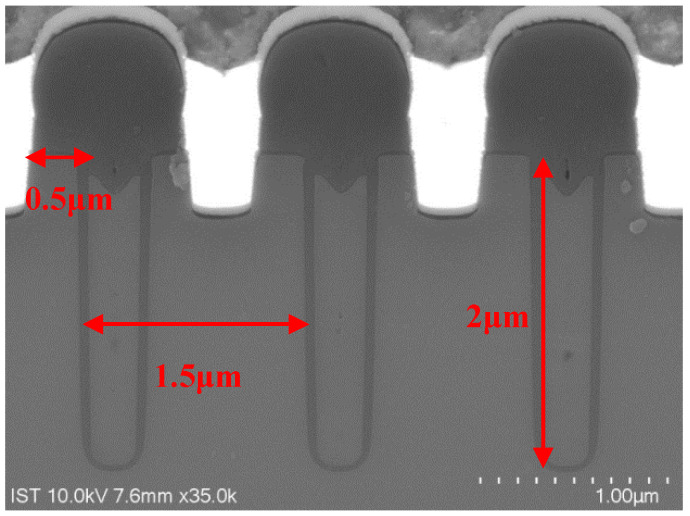
Device structure: cross-sectional SEM photograph.

The cross-sectional SEM schematic photograph of the 60 V n-channel trench VDMOS is shown in Figure 1. The devices were manufactured using 0.35 μm process. The trench structure is formed by Si trench etching, oxide growth, and poly-silicon deposition. Specifically, the gate oxide is grown after the ion doping and drive-in process to improve its quality. Table 1 lists the main parameters of the device. Because of the trench structure, the trench VDMOS exhibits a smaller cell pitch of 1.5 μm and a higher drift doping concentration of 3 × 10^16^ cm^−3^. Therefore, the devices achieve a static on-state resistance of 15 mΩ and a continuous drain current of 35 A.

The trench VDMOS devices were irradiated using a ^181^Ta ions with a linear energy transfer (LET) of 80.28 MeV·mg^−1^·cm^−2^, which were normal to the surface of the device with a beam flux of 1 × 10^4^ ions/cm^2^/s. When the total ion beam of 1 × 10^7^ ions/cm^2^ was achieved, the beam was shuttered immediately following the detection of the gate and drain leakage currents. However, when the device failed, the experiment would be terminated in advance.

**Figure 2:**
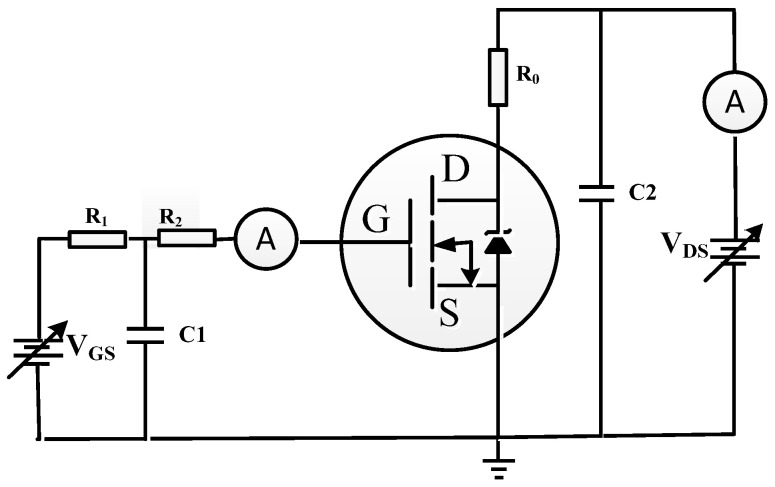
The basic test circuit for SEB and SEGR.

Figure 2 presents the basic test circuit for SEB and SEGR referring to the method 1080.1 in the MIL-STD-750-1 [21]. The gate resistor–capacitor network provides a low-pass filter to protect the gate oxide from switching transients. The drain resistor helps limit the current and voltage to prevent device failure from SEB. During the heavy-ion experiment, the drain–source voltage (*V*_DS_) was biased at 24 V or 48 V, the gate–source voltage (*V*_GS_) was biased at 0 V. The gate leakage current and the drain current were measured in real time.

## 3. Experimental Results and Analysis

**Figure 3:**
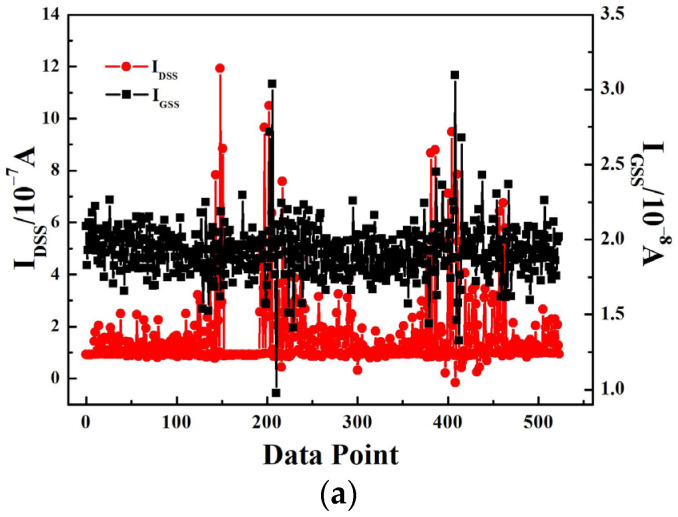

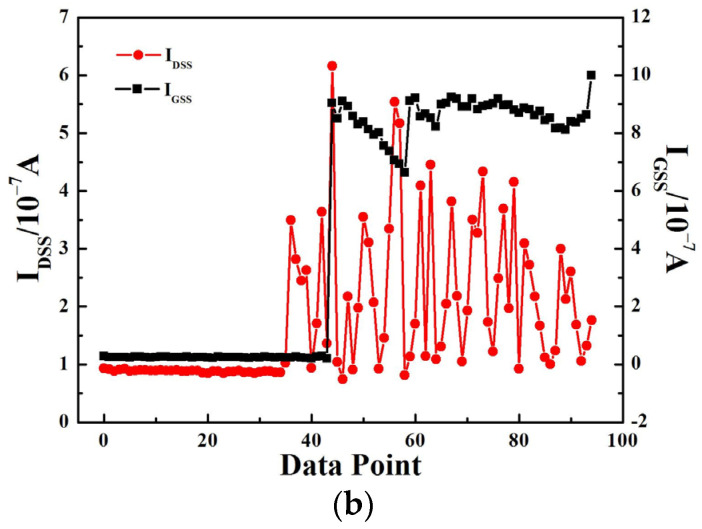
The single event test data of the trench VDMOS: (**a**) *V*_DS_ = 24 V, *V*_GS_ = 0 V; (**b**) *V*_DS_ = 48 V, *V*_GS_ = 0 V.

Figure 3a and Figure 3b show the gate leakage current and the drain current curve at *V*_DS_ = 24 V, *V*_GS_ = 0 V and *V*_DS_ = 48 V, *V*_GS_ = 0 V, respectively. As is shown in Figure 3a, the gate leakage current and the drain current had little change. This indicates that the device would not exhibit SEB or SEGR at the *V*_DS_ = 24 V and *V*_GS_ = 0 V. However, in Figure 3b, the gate leakage current increased from below 1 nA to 1 μA and exceeded the upper limit value, while the drain current varied within 1 μA. It is obviously seen that the SEGR event had occurred. After the heavy-ion experiment, the device was measured again using the AgilentB1505A and the test results show that the gate oxide of the device had suffered damage.

**Figure 4:**
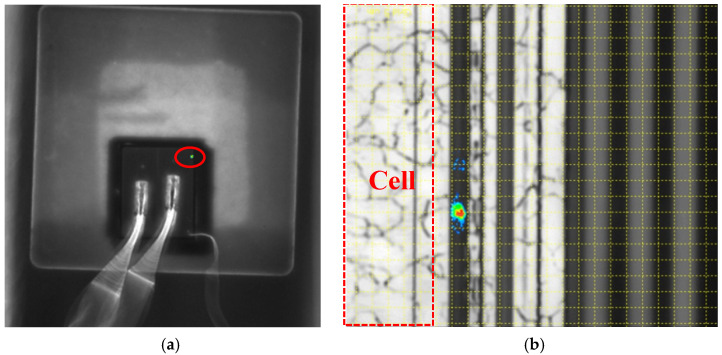
One hot spot detected by the EMMI (**a**) and the enlarged image under a microscope (**b**). The red dash frame in (**b**) represents the cell regions of the device.

**Figure 5:**
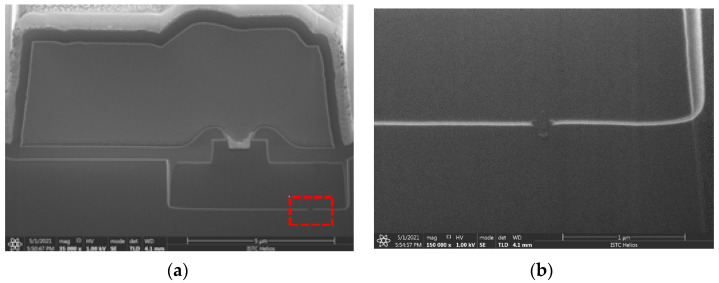
The FIB cut cross section of the hot spot by SEM (**a**) and the enlarged image of the red dash frame in (**a**) by SEM (**b**).

In order to locate the failure point of the post-irradiated trench VDMOS more accurately, an emission microscopy (EMMI) with a wavelength range from 350 nm to 1100 nm was used to detect hot spots, as shown in Figure 4. In Figure 4a, one hot spot was detected, and it can be seen clearly under a microscope. The enlarged image of the hot spot was shown in Figure 4b. By comparing it with the trench device layout, it is obviously seen that the hot pot appeared in the termination region. The red dash frame in Figure 4b represents the cell region of the device. In addition, the location of the hot spot was cut by the focused ion beam (FIB), and then the FIB-cut cross section was scanned by the scanning electron microscope (SEM), as shown in Figure 5a, the red dash frame of which was magnified as shown in Figure 5b. From Figure 5b, it is obvious that the gate oxide in the termination region had been damaged. It can be further proven that the SEGR occurred. The failure of the post-irradiated trench was caused by the SEGR in the termination region.

**Figure 6:**
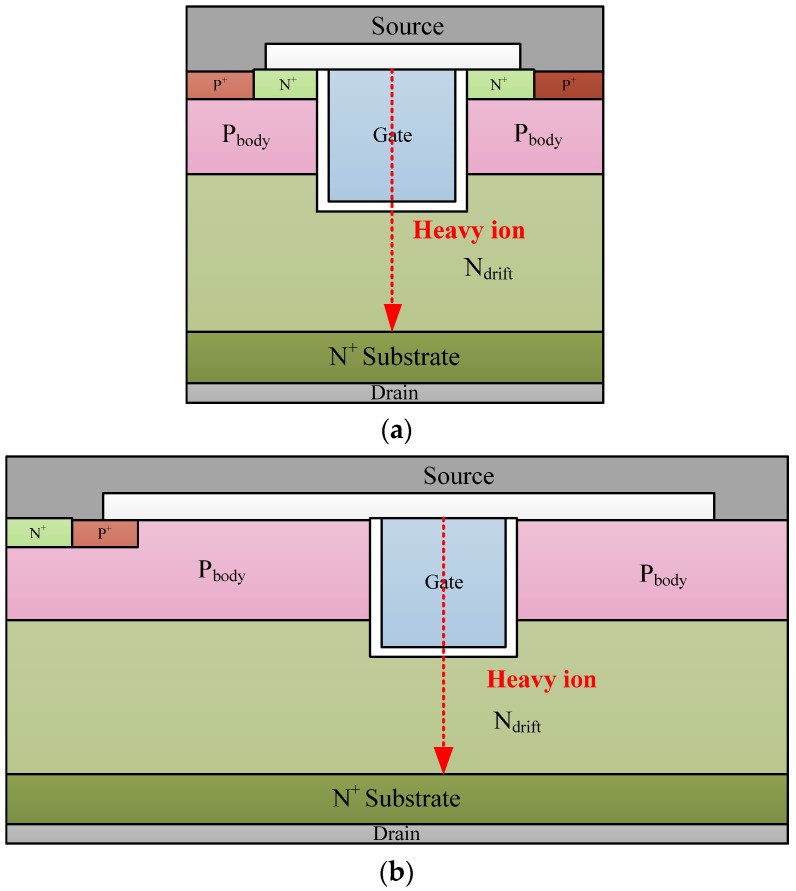
The cross section of the cell (**a**) and the termination (gate bus) (**b**). The red dotted arrows show the incident position and direction in simulation.

**Figure 7:**
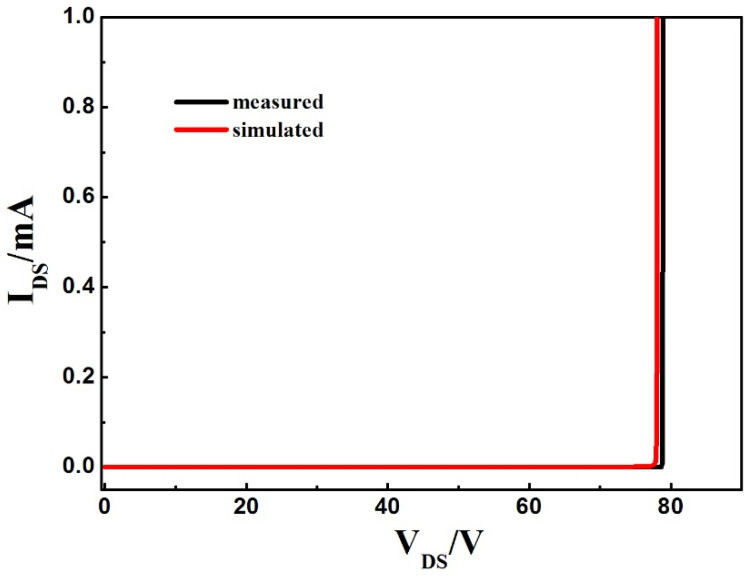
Experimental and simulated breakdown characteristics for the trench VDMOS.

To analyze the SEGR of the trench VDMOS clearly, Sentaurus TCAD simulations were performed on the trench VDMOS. The cross section of the cell and termination are shown in Figure 6a,b. The termination structure is the gate bus region used to transfer the gate signal in the cell region. The mobility model, Shockley–Read–Hall (SRH) recombination, Auger, and avalanche model were adopted in the simulation. The simulation models have been calibrated with the measured results. Figure 7 shows the simulated breakdown characteristic of the cell, which fits well with the experimental results. To mimic heavy-ion strikes, the HeavyIon model was used in simulations. The heavy ions with an LET of 80 MeV·mg^−1^·cm^−2^ were normally incident to the middle of the trench gate in the cell and termination structure. The Gaussian distribution was used in the HeavyIon model, with the radius and the length of heavy ions defined as 0.5 μm and 8 μm, respectively. The length of heavy ions was long enough to penetrate the whole drift region. The incident time of the heavy ion is 0.1 ns.

**Figure 8:**
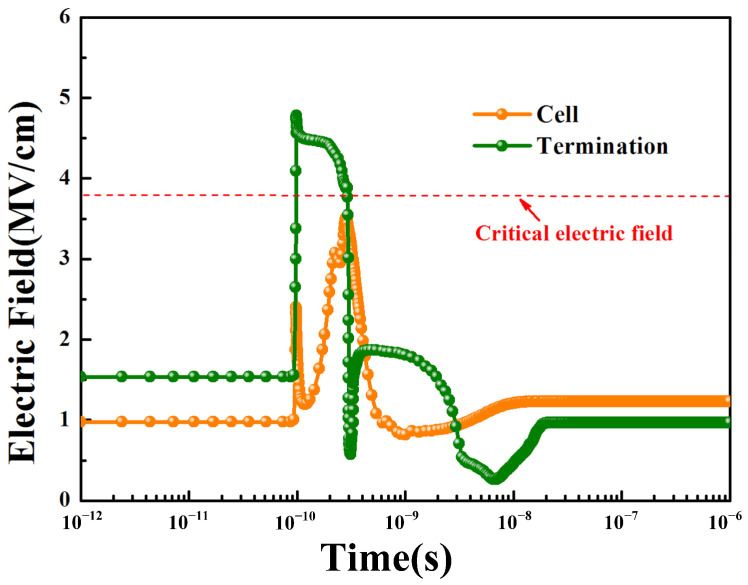
Simulated electric field of gate oxide at the position of the maximum electric field in the cell and the termination under heavy-ion strikes.

According to reference [22], the calculated critical breakdown electric field for the gate oxide is 3.76 × 10^6^ V/cm under ^181^Ta ions for triggering the SEGR. Figure 8 shows the simulated electric field of gate oxide at the position of the maximum electric field in the cell and the termination at *V*_DS_ = 40 V, *V*_GS_ = 0 V. As shown in Figure 3, the highest electric field in the termination was much larger than that of the cell. Meanwhile, the highest electric field in the cell has not reached the critical electric breakdown field, but that in the termination has far exceeded it.

**Figure 9:**
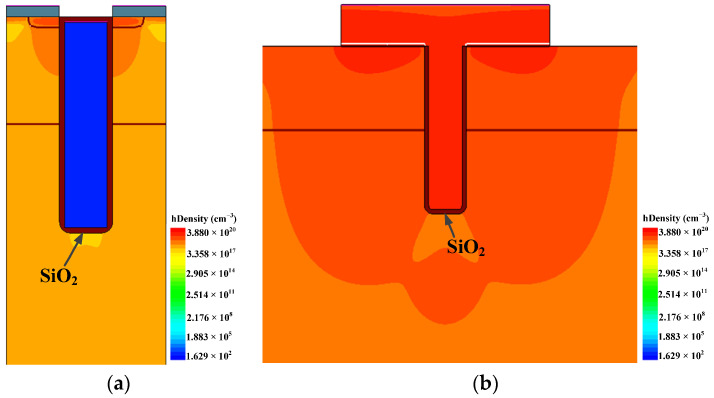
Hole density distribution near gate oxide in (**a**) the cell and (**b**) the termination at 100 ns.

During the heavy-ions strike, a large number of electron–hole pairs are generated. The electrons are swept down to the drain substrate, while the holes move to the gate when the *V*_GS_ is zero or negative and the *V*_DS_ is positive. More accumulated holes at the bottom of the gate oxide lead to a higher electric field of the gate oxide. However, the moving path of the holes in the termination is much longer than that in the cell, therefore the holes in the termination are difficult to release, which results in a higher electric field of the gate oxide. Figure 9 shows the hole density distribution near the gate oxide in the cell and the termination at 100 ns. It proves that the hole density in the termination is much higher than that in the cell.

## 4. Hardening Design and Discussion

**Figure 10:**
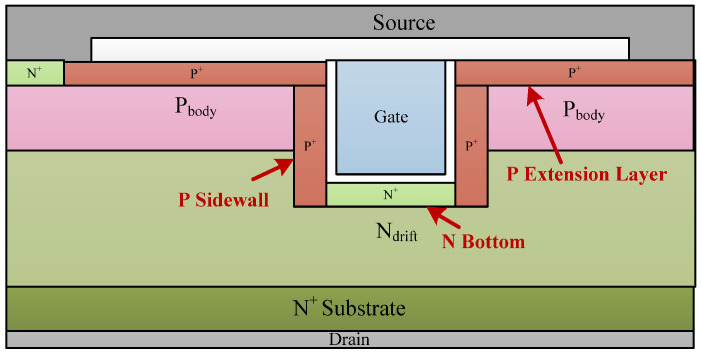
The cross section of the SEGR-hardened termination.

Figure 10 shows the cross section of the SEGR-hardened termination with multiple implantation regions of the bottom N-well region (BNW), the p-type sidewall region (SW), and the p-type extension layer (EL). The doping concentrations of BNW, SW, and EL are 5 × 10^16^ cm^−3^, 1 × 10^18^ cm^−3^, and 1 × 10^19^ cm^−3^, respectively. BNW increases the distance between the gate oxide and the hole accumulation region, which diminishes the induced electric field. Owing to the higher doping concentration of SW and EL, the resistivity of the holes’ conductive path can be obviously decreased to accelerate the holes’ release. Therefore, the accumulated holes induced by the heavy-ion strikes can be easily discharged, and the electric field of the gate oxide is decreased.

**Figure 11:**
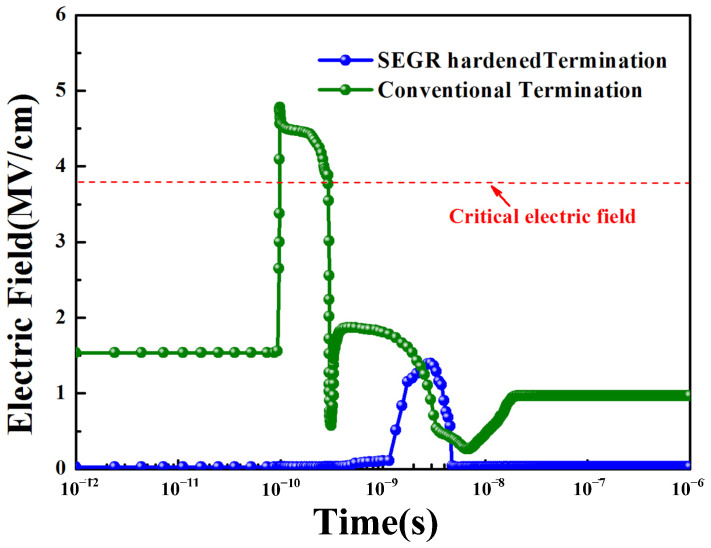
Simulated electric field of gate oxide at the position of the maximum electric field in the conventional termination and the SEGR-hardened termination with time under heavy-ion strikes.

The simulated electric field of the gate oxide at the position of the maximum electric field in the conventional termination and the SEGR-hardened termination at *V*_DS_ = 40 V, *V*_GS_ = 0 V is shown in Figure 11. It can be seen that the maximum electric field has reduced from 4.78 MV/cm in the conventional termination to 1.39 MV/cm in the SEGR-hardened termination. This means that the SEGR technique is highly effective in restraining the increase in the electric field in the gate oxide.

**Figure 12:**
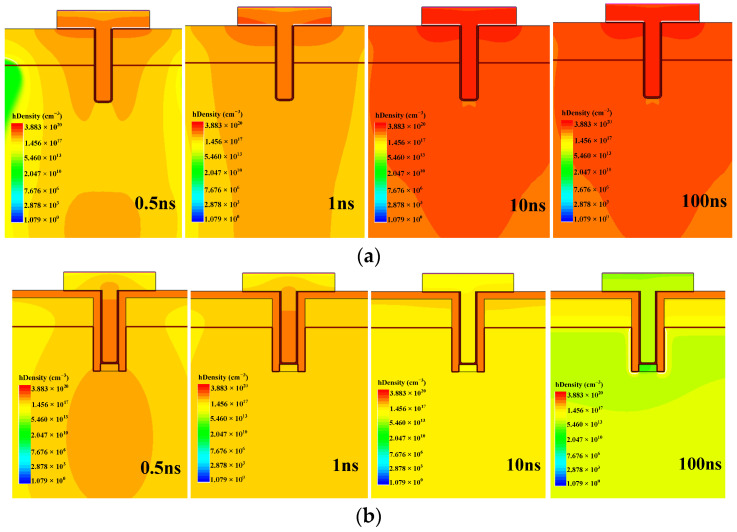
Hole density distribution near gate oxide for the conventional termination and the SEGR-hardened termination: (**a**) conventional termination; (**b**) SEGR-hardened termination.

Figure 12 shows the distribution of hole density over time of the conventional termination and the SEGR-hardened termination. It is clear that as the multiple implantation regions are introduced, the accumulated holes near gate oxide are fewer in the SEGR-hardened termination, leading to a lower electric field than the conventional termination and further ensuring the radiation reliability of the device.

**Figure 13:**
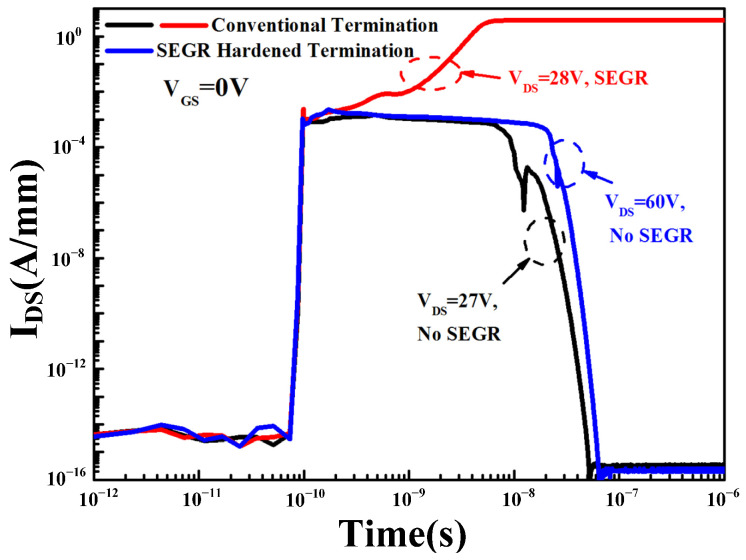
Comparison of I_DS_ when *V*_DS_ is 27 V and 28 V of the conventional termination and when *V*_DS_ is 60 V of the SEGR-hardened termination.

To further demonstrate the improvement of SEGR hardening, the SEGR-triggering criteria for the conventional termination and the SEGR-hardened termination are investigated in detail. As shown in Figure 13, it is clear seen that when *V*_GS_ is set as 0 V, the conventional termination does not trigger SEGR when *V*_DS_ is 27 V and below, and when *V*_DS_ is 28 V, SEGR occurs. Thus, the SEGR-triggering voltage (V_SEGR_) of the conventional termination is 28 V. The SEGR-hardened termination does not trigger SEGR when *V*_DS_ is 60 V and below, which can be considered to be a safe operating area (SOA); we believe that the device will not exhibit SEGR at *V*_GS_ = 0 V for 60 V trench VDMOS. Compared with the conventional termination, the SEGR-triggering critical voltage for the SEGR-hardened termination is improved by 114%.

**Figure 14:**
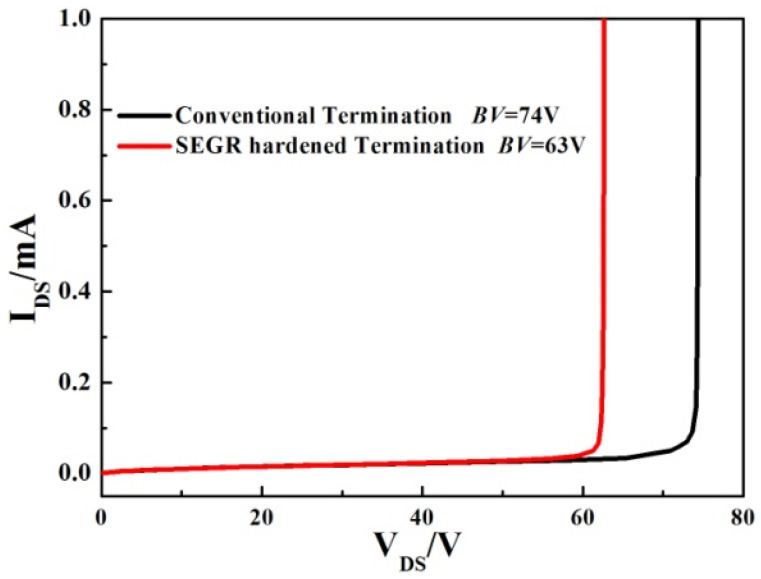
Simulated breakdown characteristic curves for the conventional termination and the SEGR-hardened termination.

The termination structure could affect the breakdown voltage of the trench VDMOS; therefore, the breakdown characteristics for the conventional termination and the SEGR-hardened termination are investigated, as is shown in Figure 14. With the introduction of multiple implantation regions for hardening, the BV is slightly decreased from 74 V to 63 V. However, compared to the low triggering voltage and failure consequences of SEGR, these small impacts of the hardening method are still acceptable.

## 5. Conclusions

In conclusion, single-event gate-rupture in trench VDMOS occurs under heavy-ions strike. With fault analysis demonstrating that the gate oxide in the termination has been damaged. The SEGR-hardened termination with multiple implantation regions of BNW, SW, and EL is proposed and simulated using Sentaurus TCAD. The multiple implantation regions result in a lower electric field in the gate oxide. Compared with the conventional termination, the SEGR-hardened termination has better radiation performance with the LET of 80.28 MeV·mg^−1^·cm^−2^.

## Figures and Tables

**Table 1:** The main parameters of the device.

Parameter	Value
Cell pitch, *W*_cell_ (μm)	1.5
Thickness of drift layer, *L*_d_ (μm)	8
Thickness of gate oxide, *t*_ox_ (μm)	0.06
Concentration of drift layer, *N*_d_ (cm^−3^)	3 × 10^16^
Concentration of P-body, *N*_p_ (cm^−3^)	1.9 × 10^17^
Concentration of P+ and N+ (cm^−3^)	1 × 10^19^

## Data Availability

Data are available on request due to restrictions, e.g., privacy or ethical.
